# Co-Occurrence of TDP-43 Mislocalization with Reduced Activity of an RNA Editing Enzyme, ADAR2, in Aged Mouse Motor Neurons

**DOI:** 10.1371/journal.pone.0043469

**Published:** 2012-08-20

**Authors:** Takuto Hideyama, Sayaka Teramoto, Kosuke Hachiga, Takenari Yamashita, Shin Kwak

**Affiliations:** 1 CREST, Japan Science Technology Agency, Kawaguchi, Saitama, Japan; 2 Department of Neurology, Graduate School of Medicine, The University of Tokyo, Bunkyo-ku, Tokyo, Japan; 3 Division for Health Promotion, The University of Tokyo, Bunkyo-ku, Tokyo, Japan; 4 Center for Disease Biology and Integrative Medicine, Graduate School of Medicine, The University of Tokyo, Bunkyo-ku, Tokyo, Japan; 5 Clinical Research Center for Medicine, International University of Health and Welfare, Ichikawa, Chiba, Japan; Okayama University, Japan

## Abstract

TDP-43 pathology in spinal motor neurons is a neuropathological hallmark of sporadic amyotrophic lateral sclerosis (ALS) and has recently been shown to be closely associated with the downregulation of an RNA editing enzyme called adenosine deaminase acting on RNA 2 (ADAR2) in the motor neurons of sporadic ALS patients. Because TDP-43 pathology is found more frequently in the brains of elderly patients, we investigated the age-related changes in the TDP-43 localization and ADAR2 activity in mouse motor neurons. We found that ADAR2 was developmentally upregulated, and its mRNA expression level was progressively decreased in the spinal cords of aged mice. Motor neurons normally exhibit nuclear ADAR2 and TDP-43 immunoreactivity, whereas fast fatigable motor neurons in aged mice demonstrated a loss of ADAR2 and abnormal TDP-43 localization. Importantly, these motor neurons expressed significant amounts of the Q/R site-unedited AMPA receptor subunit 2 (GluA2) mRNA. Because expression of unedited GluA2 has been demonstrated as a lethality-causing molecular abnormality observed in the motor neurons, these results suggest that age-related decreases in ADAR2 activity play a mechanistic role in aging and serve as one of risk factors for ALS.

## Introduction

Given that abnormal processing and mislocalization of the 43-kDa TAR DNA-binding protein (TDP-43) occurs in a variety of neurodegenerative diseases, including amyotrophic lateral sclerosis (ALS) and frontotemporal lobar degeneration (FTLD-TDP) [1], [2], much effort has been made to understand the molecular bases of the abnormalities that occur in TDP-43 proteinopathies. TDP-43 pathology is frequently observed in spinal motor neurons of patients with sporadic ALS, implicating TDP-43 pathology as a pathological hallmark of ALS.

Recent studies on the spinal cords of patients with sporadic ALS have demonstrated that TDP-43 pathology exclusively appears in motor neurons with reduced expression of the RNA editing enzyme, adenosine deaminase acting on RNA 2 (ADAR2) [3]. ADAR2 catalyzes the adenosine-to-inosine (A-to-I) conversion of pre-mRNA, including the glutamine/arginine (Q/R) site in the pre-mRNA of AMPA receptor subunit 2 (GluA2), a subunit of the L-a-amino-3-hydroxy-5-methyl-4-isoxazolepropionic acid (AMPA) receptors [4]. In the motor neurons of patients with sporadic ALS, the A-to-I conversion at the GluA2 Q/R site is inefficient, and approximately half of the motor neurons express the Q/R site-unedited GluA2 in the majority of ALS cases, rendering the expression of unedited GluA2 as another disease-specific molecular abnormality of sporadic ALS [5], [6]. Importantly, the ADAR2-lacking motor neurons underwent slow cell death specifically because of their failure to edit the Q/R site of GluA2, as observed in the conditional ADAR2 knockout (ADAR2^flox/flox^/VAChT-Cre.Fast, or AR2) mice [7]. This indicates that failure of the GluA2 Q/R site resulting from reduced ADAR2 expression is involved in the pathogenesis of sporadic ALS. Because the AMPA receptors that contain the Q/R site-unedited GluA2 are permeable to Ca^2+^, the neurotoxicity mediated by Ca^2+^-permeable AMPA receptors containing unedited GluA2 is likely the mechanism involved in the degeneration of motor neurons in sporadic ALS patients [8], [9].

Aging is a risk factor in many neurodegenerative disorders, including ALS. The majority of patients with ALS manifest initial symptoms after middle age, and the incidence of ALS increases with age [10], [11]. In addition, the disease progression after onset is faster in elderly patients compared with younger patients [11]. TDP-43 pathology is observed more frequently in elderly patients than in younger patients and in neurologically normal elderly people, although this is not frequent (approximately 3%) [12], [13]. The close association between TDP-43 pathology and reduced ADAR2 expression in ALS motor neurons suggests a molecular link between the two [3], and investigation of the molecular link in the motor neurons of elderly subjects will provide insight into the molecular mechanisms that underlie the age-related acceleration of ALS symptoms. In this study, we demonstrated that TDP-43 mislocalization occurred in association with the reduction of ADAR2 activity in the motor neurons of aged mice.

## Materials and Methods

### Ethics Statement

All of the studies were performed in accordance with the Declaration of Helsinki, the Guideline of Animal Studies of the University of Tokyo and the NIH. The animal handling committee at the University of Tokyo also approved the experimental procedures performed in this study.

### Case Material

We used C57BL/6J mice at one day, and 1 week, 2, 6, 12, 15, and 26 months of age (n = 3 for each group).

### Isolation of AH and Motor Neurons Tissue

Anterior horns of the spinal cord (AH) and motor neurons in the AH were isolated from frozen spinal cord tissues with a laser microdissection system (Leica AS LMD, Leica Microsystems) as previously described [6], [7], [14]. Frozen AH tissues were dissected separately from the posterior horns and white matter from 20-µm-thick cervical cord sections under a binocular. Whole spinal cord was isolated from the mice at 1 day and 7 days of age. For the analysis on the MN tissues, we dissected five motor neurons with diameter larger than 20 µm in the lateral area of each AH, and collected them together into a single test tube containing 200 µl of TRIZOL Reagent. We dissected motor neurons from 4 AH of each mouse.

### Analysis for Editing Efficiency at A–I Sites

Total RNA was isolated from the dissected tissues, and first-strand cDNA was synthesized and then treated with DNAase I (Invitrogen) as previously described [7], [14]. Editing efficiencies at the Q/R sites in GluA2 mRNAs were calculated by quantitative analyses of the digests of RT-PCR products with *BbvI* as previously described [5], [6], [7], [15]. In brief, 2 µl of cDNA were subjected to first PCR in duplicate in a reaction mixture of 50 µl containing 200 mM each primer, 1 mM dNTP Mix (Eppendorf AG), 5 µl of 10× PCR buffer and 1 µl of Advantage 2 Polymerase mix (BD Biosciences Clontech). The PCR amplification began with a 1-min denaturation step at 95°C, followed by 40 cycles of denaturation at 95°C for 10 s, annealing at 60°C for 30 s and extension at 68°C for 40 s. Nested PCR was conducted on 2 µl of the first PCR product under the same conditions with the exception of the annealing temperature (58°C). Primer pairs used for each PCR were used previous report [7] (Table S1). After gel purification using the Zymoclean Gel DNA Recovery Kit according to the manufacturer’s protocol (Zymo Research), an aliquot (0.5 µg) was incubated with *BbvI* (New England BioLabs) at 37°C for 12 h. The PCR products originating from Q/R site-edited GluA2 mRNA had one intrinsic restriction enzyme recognition site, whereas those originating from unedited mRNA had an additional recognition site. Thus, restriction digestion of the PCR products originating from edited GluA2 mRNA should produce different numbers of fragments (two bands at 219- and 59-bp) from those originating from unedited GluA2 mRNA (three bands at 140-, 79- and 59-bp). As the 59-bp band would originate from both edited and unedited mRNA, but the 219-bp band would originate from only edited mRNA, we quantified the molarity of the 219- and 59-bp bands using the 2100 Bioanalyzer (Agilent Technologies) and calculated the editing efficiency as the ratio of the former to the latter for each sample (Figure S1) [7]. With similar methods, we calculated the editing efficiencies at the lysine/glutamate (K/E) sites in cytoplasmic fragile × mental retardation protein interacting protein 2 (CYFIP2) mRNA and pre-mRNA (Table S1) [5], [6], [14], [15], [16], [17], [18]. Following restriction enzymes were used for restriction digestion of the respective A-to-I sites; *MseI* (New England BioLabs) for the K/E site. Primer pairs used for each PCR and sizes of restriction digests of PCR products were indicated in Table S1.

### Immunohistochemistry

Under deep anesthesia with isoflurane, mice (6, 15, and 26 months of age, n = 3 for each group) were transcardially perfused with 3% paraformaldehyde and 1% glutaraldehyde in phosphate-buffered saline (PBS). The spinal cords were removed and immersed in serially increasing concentrations of a sucrose-PBS solution (final sucrose concentration of 30%). Immunohistochemical procedure was carried out on 10-µm-thick sections, which were cut with a cryostat (Model HM500 O; MICROM) after rapid freezing with dry ice. The sections mounted on slides were then washed with PBS and incubated with the primary antibody for 1 hour at room temperature. Polyclonal anti-RED 1 (Exalpha Biologicals, Inc; at a dilution of 1∶100) and rabbit polyclonal anti-TARDBP (Protein-Tech Group, Inc; at a dilution of 1∶3,000) were used [3]. After 3 washes with PBS, 5 min at room temperature, samples were incubated for 30 min with donkey anti-sheep IgG conjugated to the fluorescent Alexa 555 dye (Invitrogen Molecular Probe; at a dilution of 1∶400) or chicken anti- rabbit IgG conjugated to the fluorescent Alexa 488 dye (Invitrogen Molecular Probe; at a dilution of 1∶400) in PBS containing the fluorescent nuclear probes TOPRO3 (Invitrogen Molecular Probe; at a dilution of 1∶250). After 3 washes, samples were mounted in Vectashield Medium (Vector Laboratories Inc, Abcys, Paris, France) for viewing with a fluorescence microscope (BIOREVO BZ-9000; Keyence Corp, Osaka, Japan).

### Morphological Observation

Sections of the fifth lumbar (L5) spinal cord segment were sequentially immunostained for ADAR2 and TDP-43 using the immuno-fluorescence system. ADAR2-positive and -negative motor neurons were separately counted among those with diameters larger than 20 µm (short axis) in ten sections for each mouse. The number of TDP-43-positive motor neurons in the ventral gray matter (ventral to the line running though the ventral edge of the central canal) was counted in ten L5 sections for each mouse.

### Quantitative PCR

The expression levels of ADAR2 and GluA2 were measured using the LightCycler system (Roche Diagnostics, Indianapolis, IN) [5], [6], [15]. To prepare an internal standard for quantitative PCR, gene-specific PCR products of approximately 1 kb in length were amplified from human cerebellar cDNA with the same primer pairs as previously reported (Table S2) [5], [6], [7], [15]. Each cDNA sample was amplified in a reaction mixture (20 µl total volume) composed of 10 µl of 2× LightCycler 480 Probes Master Roche (Roche Diagnostics), 0.5 mM of each primer set and 0.1 mM probes (Universal Probe Library Set, Human, Roche Diagnostics) (Table S3).

### Statistics

Differences and correlations between two groups were evaluated using Mann-Whitney U-test with SPSS software (version 15; SPSS Inc., Chicago, IL). Moreover, differences between six or seven groups were evaluated using repeated ANOVA with GraphPad Prism version 5 (GraphPad Software, Inc., La Jolla, CA). Differences were considered statistically significant with P<0.01 and highly significant with P<0.001.

## Results

**Figure 1:**
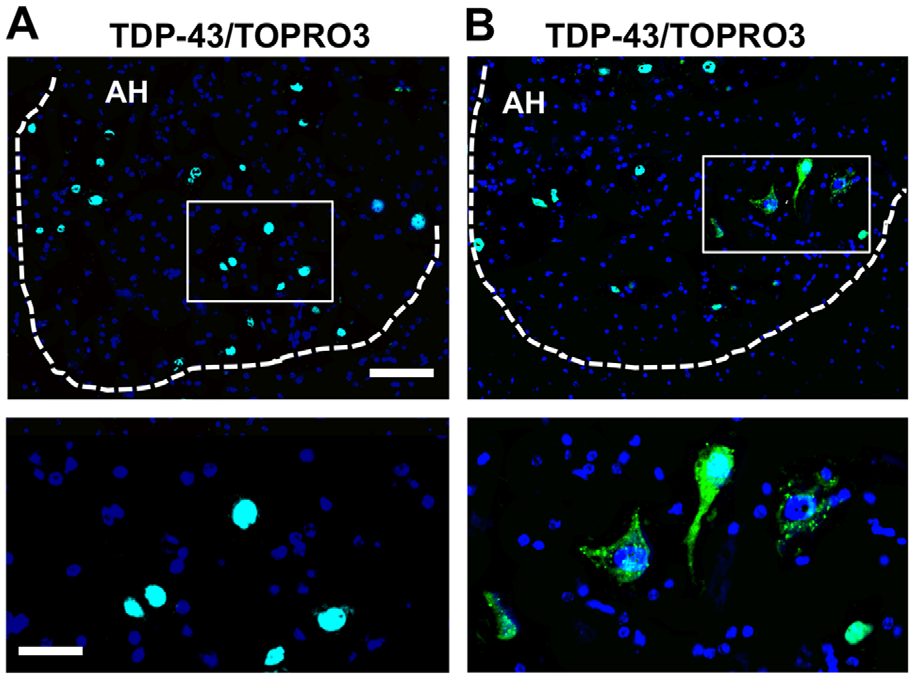
Abnormal localization of TDP-43 in aged mouse spinal cords. TDP-43 (green) immunoreactivity is shown in the anterior horn (AH) cells of mouse spinal cords. (**A**) TDP-43 immunoreactivity (white arrows) is confined to the nuclei in the neurons, including the large motor neurons of the mouse at 6 months of age. (**B**) TDP-43 is absent from the nucleus and mislocalizes into the cytoplasm in the mouse at 26 months of age. Each frame area is enlarged in the inset to show the subcellular localization of TDP-43. TOPRO3 (blue) stains the nucleolus. Scale bars: 120 µm. Inset: 40 µm.

Mice that were less than one year old exhibited nuclear TDP-43 immunoreactivity in all neurons, including motor neurons (Figure 1A). In contrast, TDP-43 immunoreactivity was less robust in the nucleus and demonstrated abnormal cytoplasmic staining in the motor neurons of aged mice that were 15 months of age (Figure 1B).

**Figure 2:**
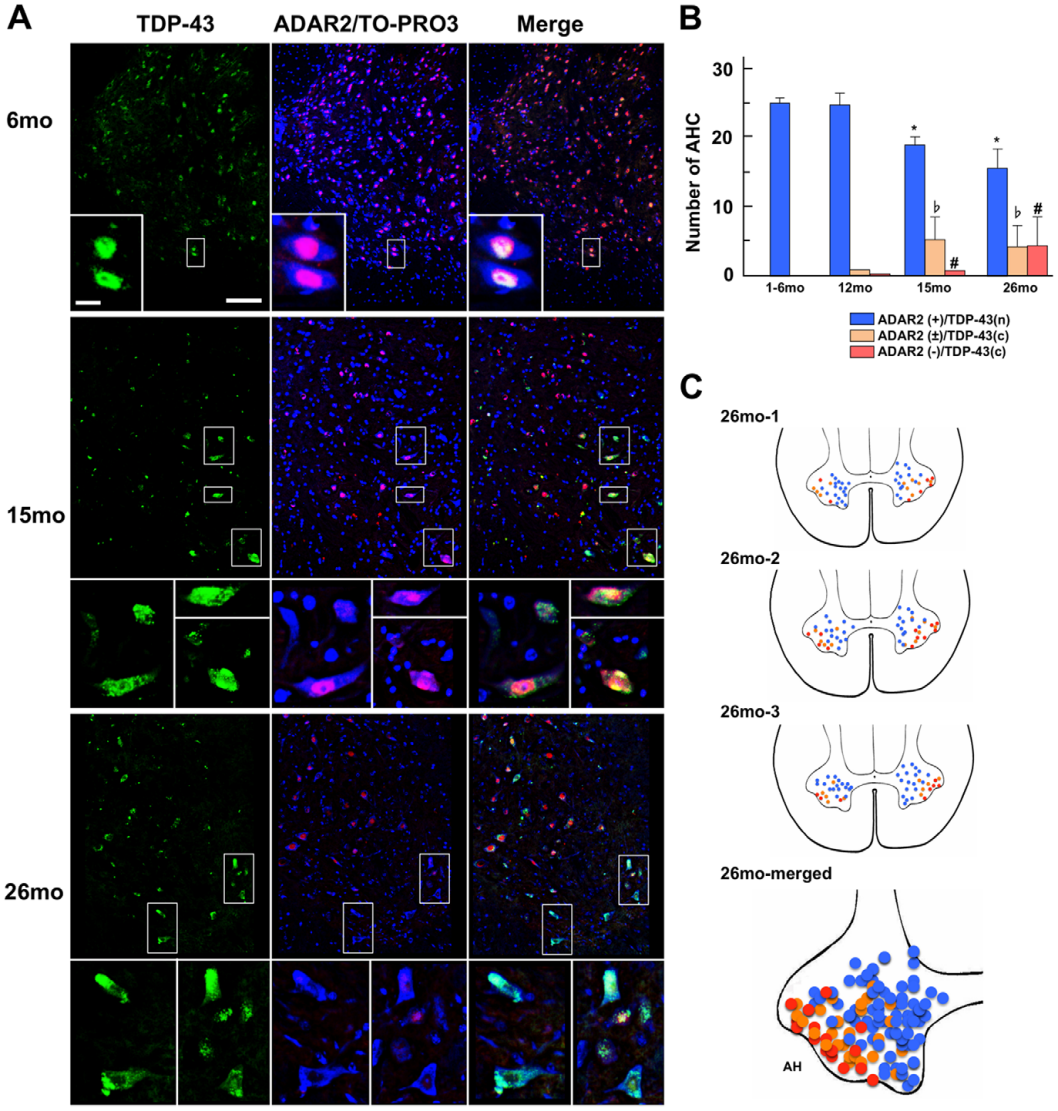
Motor neurons exhibiting an age-related increase of abnormal TDP-43 localization and reduced ADAR2 immunoreactivity. (**A**) Representative confocal images of mouse anterior horns of the spinal cord (AH) at 6 months (6 mo), 15 months (15 mo) or 26 months (26 mo) of age. Three mice at each age were investigated. The frame area is enlarged in the inset to show TDP-43 (green) and ADAR2 (red)/TOPRO3 (blue) in the motor neurons. Both the ADAR2 and TDP-43 immunoreactivities are confined to the nuclei of all mouse motor neurons at 6 months of age. Scale bars: 100 µm. Inset: 20 µm. (**B**) Numbers (means ± SEMs) are shown for motor neurons with normal ADAR2- and TDP-43-immunoreactivity (blue columns), those with low ADAR2-immunoreactivity in the nucleus and mislocalization of TDP-43 (beige columns), and those that lack ADAR2-immunoreactivity in the nucleus (pink columns) in wild-type mice at different months (m) of age (1–6, 12, 15, and 26 m; n = 3 each). The number of motor neurons with normal immunoreactivity is significantly decreased (*p<0.01), whereas the number of motor neurons with abnormal ADAR2/TDP-43-immunoreactivity is significantly increased after 15 months of age compared with 12 months of age (^♭^ *p*<0.01, ^#^ *p*<0.01, Mann-Whitney U-test). (**C**) The positions of the motor neurons with abnormal immunoreactivity in the mouse AH at 26 months (n = 3). The colors (blue, beige, and pink) representing the motor neurons with different immunohistochemical patterns are the same as in Figure 2B. All of the motor neurons examined in the 3 mice at 26 months of age are plotted in the same figure as the AHs. Note that the motor neurons with abnormal immunoreactivity (beige and pink) are localized in the lateral areas of the AHs, suggesting that they are the fast fatigable motor neurons.

Double immunostaining for TDP-43 and ADAR2 revealed strong immunoreactivity to both TDP-43 and ADAR2 in the neuronal nuclei in young mice but low or absent immunoreactivity to ADAR2 and cytoplasmic mislocalization of TDP-43 in the motor neurons of aged mice (Figure 2A). There was a strict correlation between the abnormal TDP-43 localization and the reduced or absent ADAR2 immunoreactivity in the nuclei of these motor neurons. ADAR2 immunoreactivity was decreased, and TDP-43 expression was abnormally localized from the nuclei to the cytoplasm in some motor neurons at 15 and 26 months of age. The mislocalization of TDP-43 was observed only in the motor neurons with low or absent ADAR2 immunoreactivity. The number of motor neurons with abnormal immunoreactivity increased in an age-dependent manner. In particular, this increase was observed most robustly after 15 months of age. In addition, at 26 months of age, approximately 15% of the motor neurons were devoid of ADAR2 nuclear immunoreactivity (Figure 2B). Based on the large size and expression pattern in the lateral AHs (Figure 2C), these motor neurons are most likely fast fatigable (FF) motor neurons [19], [20], [21].

**Figure 3:**
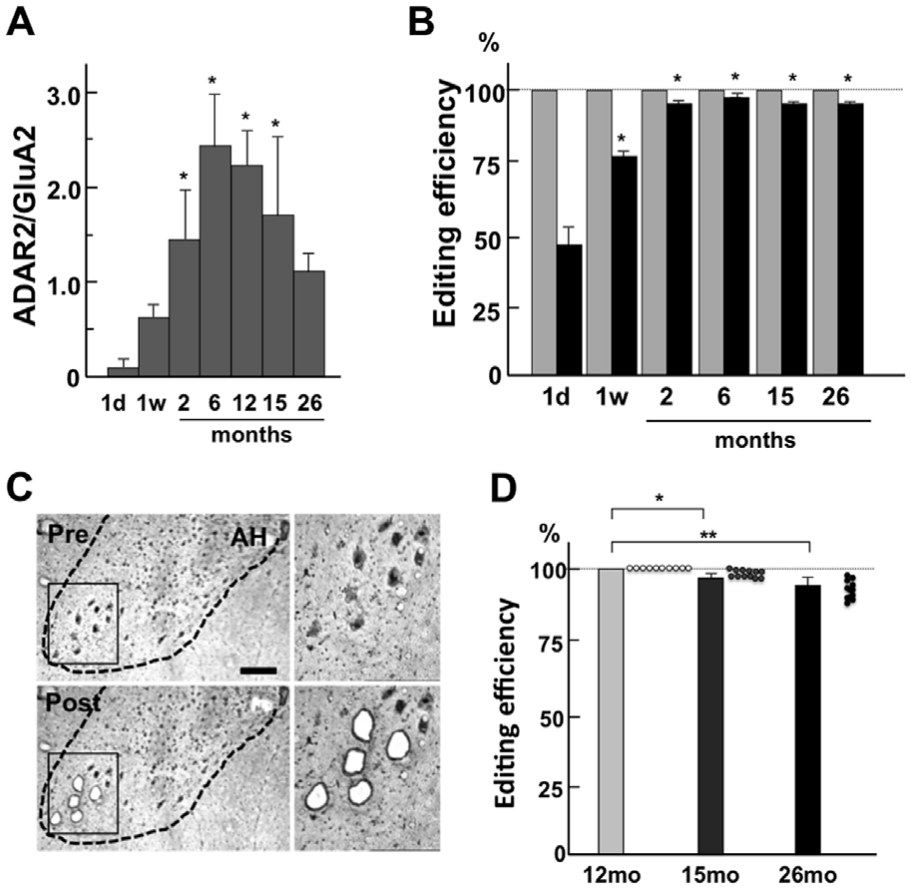
Age-related changes in ADAR2 activity in the AHs and motor neurons. (**A**) The relative abundance of the ADAR2 mRNA in the anterior horns of the spinal cord (AHs) is shown at the GluA2 mRNA base (n = 3 for each age). The expression level of ADAR2 mRNA developmentally increases and decreases in an age-dependent manner from 26 months of age. The columns and bars represent means ± SEMs. The data were analyzed using repeated ANOVA. Contrast tests were used to compare the means of the ADAR2 activities between the groups after 2 months of age. The values in the four groups (2, 6, 12, and 15 months of age) were significantly higher than the value in the group at 26 months of age (*p<0.01). (**B**) The editing efficiency at the GluA2 Q/R site was maintained at 100% across the ages, whereas the editing efficiency at the CYFIP2 K/E site developmentally increased in the AHs. The data were analyzed using repeated ANOVA. Contrast tests were used to compare the means of the editing efficiencies between the groups. The values in the five groups (1 week of age, 2, 6, 12, and 15 months of age) were significantly higher than the value in the group at one day of age (*p<0.01). (**C)** Mouse AHs at 26 months of age are demarcated with dotted lines. A magnified view of the boxed region is shown in the right panel. The figures show the AHs pre- and post-dissection of the five motor neurons in the lateral AHs. Dissection was performed with a laser microdissector. The sections were stained with 0.1% toluidine blue. Scale bars: 100 µm. (**D**) The editing efficiencies at the GluA2 Q/R site in the lysates of five motor neurons obtained from mice at 26 months (26 mo), 15 months (15 mo), and 12 months (12 mo) of age. The editing efficiencies for the 26 mo group (95.3% ±2.6%; n = 12) were significantly lower than those for the 12 mo group (100%; n = 10) (**p<0.001) and 15 mo group (98.1% ±0.7%; n = 10) (*p<0.005). Each circle represents the editing efficiency at the GluA2 Q/R site in the lysates of five motor neurons from the 26 mo (black), 15 mo (gray), and 12 mo groups (white). The columns and bars represent the means ± SEMs. The data were analyzed using a Mann-Whitney U-test.

Next, we examined the age-dependent changes in the activity of ADAR2 in the lysates of the AHs. The expression level of ADAR2 mRNA relative to the GluA2 mRNA increased during development and decreased in the aged mice (Figure 3A). Consistent results were obtained when we normalized the ADAR2 mRNA level with β-actin mRNA (Figure S2). Because ADAR2 specifically catalyzes the A-to-I conversion at the Q/R site of the GluA2 pre-mRNA and the K/E site of the CYFIP2 pre-mRNA [17], [18], [22], we examined the proportions of these mRNAs that were edited at the corresponding A-to-I position of the total mRNA (referred to as the editing efficiency). The editing efficiency at the GluA2 Q/R site was 100% across the ages examined, and we observed a developmental increase in the editing efficiency at the CYFIP2 K/E site (Figure 3B). These results indicated that the developmental increase in the ADAR2 expression correlated with the ADAR2 activity at the CYFIP2 K/E site. However, an age-dependent reduction in ADAR2 expression may not be sufficiently extensive to decrease the editing efficiency at the CYFIP2 K/E site or the GluA2 Q/R site in the AH.

Because the motor neurons with mislocalized TDP-43 were found only in the lateral AH (Figure 3C), we investigated the ADAR2 activity at the Q/R site of GluA2 mRNAs in the lysates of five laser-captured motor neurons in the lateral AHs (Figure 3C). The editing efficiency of the GluA2 Q/R site in the lysates of laterally located large motor neurons was decreased in an age-dependent manner; 100% (mice at 12 months, n = 10), 98.1% ±0.8% (15 months, n = 12, *p*<0.005) and 95.3% ±2.6% (26 months, n = 10, *p*<0.01) (Figure 3D). In contrast, the RNA editing at the GluA2 Q/R site in the lysates of neurons in other areas of the spinal cord was preserved even in mice at 26 months of age (99.7% ±0.78%). It is likely, therefore, despite the widespread reduction in the ADAR2 mRNA expression level in the aged mouse spinal cord, the age-dependent reduction in ADAR2 activity reached at a level insufficient to edit the Q/R site of all of the GluA2 mRNA only in the motor neurons in the lateral AHs of the elderly mice.

## Discussion

We demonstrated that both TDP-43 and ADAR2 localized normally to the nuclei of the motor neurons of young and adult mice. In contrast, TDP-43 mislocalized from the nucleus to the cytoplasm in association with a reduction of ADAR2 immunoreactivity in the nuclei of motor neurons in an age-dependent manner. These two abnormalities were always observed in the same motor neurons, which were located in the lateral AHs. In addition, the number of abnormal TDP-43-expressing motor neurons increased with aging, specifically after 15 months of age. ADAR2 expression also decreased in an age-dependent manner; however, after examination of the AH lysates, we determined that this occurred without a significant reduction in the editing efficiency of the ADAR2-mediated A-to-I positions. Interestingly, the laterally located motor neurons exhibited a Q/R site-unedited GluA2 mRNA with abnormal TDP-43 and ADAR2 immunoreactivity in the aged mice. Taken together, these results suggest that ADAR2 expression is downregulated in an age-dependent fashion in murine motor neurons, and mislocalization of TDP-43 occurs in association with the expression of Q/R site-unedited GluA2 mRNA but not with a reduction of ADAR2 mRNA.

The laterally located large motor neurons in which ADAR2 downregulation and mislocalization of TDP-43 were observed are most likely fast fatigable (FF) motor neurons that extend fast-conducting axons [19], [20], [21]. The twitch force of fast motor units in the electromyogram is affected earliest in patients with sporadic ALS [23], and FF motor units degenerate early compared with motor neurons that innervate slow muscles or those that are involved in eye movement and pelvic sphincter control in ALS patients and aged subjects [24], [25]. Moreover, FF motor neurons are selectively vulnerable to stress caused by axonal injury [26] and aging [27], [28] in wild-type mice, exhibiting a 35% decrease in the number of FF motor units and a 15% decrease in size in the medial gastrocnemius muscles [29], [30]. It is of interest that the laterally located large motor neurons (here we arbitrarily call as FF motor neurons) are more susceptible to age-related degeneration and other unfavorable conditions compared with other types of motor neurons in humans and rodents.

ADAR2 mediates the A-to-I conversion at various positions in numerous pre-mRNAs that are expressed in the central nervous systems of mammals and humans, including the Q/R site of GluA2 and the K/E site of CYFIP2 [17], [18], [31]. The GluA2 Q/R site-editing is 100% preserved throughout life and as early as the embryonic stage in mammalian [32], [33] and human brains [34]. In addition, neurons can express sufficient amounts of ADAR2 to edit all of the GluA2, which indicates that a mild reduction in the expression level of ADAR2 does not reduce the extent of the GluA2 Q/R site-editing in motor neurons [15]. Therefore, the expression level of the ADAR2 mRNA relative to the GluA2 mRNA [15] and the extent of CYFIP2 K/E site-editing [17], [18], [31] have been used as a marker for ADAR2 activity in mammalian brains and spinal cords. The results of this study demonstrated that the change in the extent of the A-to-I conversion at the CYFIP2 K/E site correlated with the developmental changes observed in the ADAR2 mRNA expression in the murine AHs. One previous study suggested an age-related reduction in the ADAR2 activity in the human brain after measuring the editing efficiency of the K/E site of CYFIP2 mRNA [35]; however, age-related changes in either the editing efficiency at the GluA2 Q/R site or the expression level of ADAR2 mRNA were not reported.

The absence of ADAR2 activity is not injurious to mouse motor neurons provided that the A-to-I conversion at the GluA2 Q/R site is complete [7]. However, all of the motor neurons slowly degenerate when they express the Q/R site-unedited GluA2 mRNA regardless of their expression levels in conditional ADAR2 knockout mice (AR2 and HeteroAR2) [7], [8]. Therefore, FF motor neurons in aged mice expressing unedited GluA2 will most likely die, albeit in a small proportion (less than 10%), although the time in which they undergo cell death is determined stochastically [8]. TDP-43 mislocalization was observed exclusively in the FF motor neurons that exhibited low or absent ADAR2 immunoreactivity. The expression level of the ADAR2 mRNA consistently decreased in the aged mouse AH, indicating that the expression of the Q/R site-unedited GluA2, rather than a reduction of ADAR2 activity *per se*, is associated with TDP-43 mislocalization. The association of TDP-43 pathology and reduced ADAR2 in aged mouse motor neurons further supports a molecular link between TDP-43 and reduced ADAR2 in ALS motor neurons [3]. Given that the reduction in the expression level of ADAR2 mRNA begins at the preclinical stage in the motor neurons of sporadic ALS patients [3], age-dependent downregulation of ADAR2 activity may accelerate the expression of the Q/R site-unedited GluA2, thereby playing a role in the age-related increase in the incidence of ALS and the acceleration of the course of disease in ALS patients [3]. We cannot exclude the possibility, although unlikely, that ADAR2 downregulation and TDP-43 pathology are indirectly linked and appear more or less by chance. If this hypothesis is correct, TDP-43 pathology in neurological diseases other than ALS might also be explained by a dysregulation of Ca^2+^ signaling. Moreover, the selective appearance of TDP-43 pathology in limited subsets of neurons and glial cells might result from the cell type-dependent differences in the vulnerability to Ca^2+^ dysregulation [7], [36], [37].

ADAR2 activity is decreased in an age-dependent manner in mouse motor neurons, particularly in the FF motor neurons, and further reduction induces TDP-43 pathology via the expression of the Q/R site-unedited GluA2. The age-related decrease of ADAR2 activity may make motor neurons more vulnerable to Q/R site-unedited GluA2-mediated ALS pathology in elderly individuals, implicating aging as a risk factor for ALS. Thus, an understanding of the underlying mechanisms of TDP-43 pathology will lead to the development of preventive or protective therapies for ALS patients.

## Supporting Information

Figure S1 **Analysis with a 2100 Bioanalyzer and examples of chromatogram.** (**A**) The PCR products originating from Q/R site-edited GluA2 mRNA had one intrinsic restriction enzyme recognition site (black arrow), whereas those originating from unedited mRNA had an additional recognition site (gray arrow). (**B**) Restriction digestion of the PCR products originating from edited GluA2 mRNA should produce different numbers of fragments (two bands at 219-bp and 59-bp) from those originating from unedited GluA2 mRNA (three bands at 140-bp, 79-bp and 59-bp). As the 59-bp band would originate from both edited and unedited mRNA, but the 219-bp band would originate from only edited mRNA, we quantified the molarity of the 219- and 59-bp bands using the 2100 Bioanalyzer (Agilent technologies Japan, Tokyo) and calculated the editing efficiency as the ratio of the former to the latter for each sample.
(TIF)

Figure S2 **Age-related changes in ADAR2 activity in the AHs.** The relative abundance of the ADAR2 mRNA in anterior horns (AHs) is shown at the β-actin mRNA base (n = 3 for each age). The expression level of ADAR2 mRNA developmentally increases and decreases in an age-dependent manner from 26 months of age. The columns and bars represent means ± SEMs. The data were analyzed using repeated ANOVA. Contrast tests were used to compare the means of the ADAR2 activities between the groups after 2 months of age. The values in the four groups (2, 6, 12, and 15 months of age) were significantly higher than the value in the group at 26 months of age (*p<0.01).
(TIF)

Table S1 **Primers for PCR, RT-PCR and restriction digestion of PCR products.**
(DOC)

Table S2 **Internal standard for quantitative PCR.**
(DOC)

Table S3 **Probes and primers for Real-Time PCR.**
(DOC)

## References

[1] Arai T, Hasegawa M, Akiyama H, Ikeda K, Nonaka T, et al (2006) TDP-43 is a component of ubiquitin-positive tau-negative inclusions in frontotemporal lobar degeneration and amyotrophic lateral sclerosis. Biochem Biophys Res Commun 351: 602–611. 17084815 10.1016/j.bbrc.2006.10.093

[2] Neumann M, Sampathu DM, Kwong LK, Truax AC, Micsenyi MC, et al (2006) Ubiquitinated TDP-43 in frontotemporal lobar degeneration and amyotrophic lateral sclerosis. Science 314: 130–133. 17023659 10.1126/science.1134108

[3] Aizawa H, Sawada J, Hideyama T, Yamashita T, Katayama T, et al (2010) TDP-43 pathology in sporadic ALS occurs in motor neurons lacking the RNA editing enzyme ADAR2. Acta Neuropathol 120: 75–84. 20372915 10.1007/s00401-010-0678-x

[4] Higuchi M, Maas S, Single FN, Hartner J, Rozov A, et al (2000) Point mutation in an AMPA receptor gene rescues lethality in mice deficient in the RNA-editing enzyme ADAR2. Nature 406: 78–81. 10894545 10.1038/35017558

[5] Takuma H, Kwak S, Yoshizawa T, Kanazawa I (1999) Reduction of GluR2 RNA editing, a molecular change that increases calcium influx through AMPA receptors, selective in the spinal ventral gray of patients with amyotrophic lateral sclerosis. Ann Neurol 46: 806–815. 10589532 10.1002/1531-8249(199912)46:6<806::aid-ana2>3.0.co;2-s

[6] Kawahara Y, Ito K, Sun H, Aizawa H, Kanazawa I, et al (2004) Glutamate receptors: RNA editing and death of motor neurons. Nature 427: 801. 14985749 10.1038/427801a

[7] Hideyama T, Yamashita T, Suzuki T, Tsuji S, Higuchi M, et al (2010) Induced loss of ADAR2 engenders slow death of motor neurons from Q/R site-unedited GluR2. J Neurosci 30: 11917–11925. 20826656 10.1523/JNEUROSCI.2021-10.2010 PMC6633551

[8] Hideyama T, Kwak S (2011) When does ALS start? ADAR2-GluA2 hypothesis for the etiology of sporadic ALS. Front Mol Neurosci. 10.3389/fnmol.2011.00033 PMC3214764 22102833

[9] Kwak S, Kawahara Y (2005) Deficient RNA editing of GluR2 and neuronal death in amyotropic lateral sclerosis. J Mol Med 83: 110–120. 15624111 10.1007/s00109-004-0599-z

[10] Haverkamp LJ, Appel V, Appel SH (1995) Natural history of amyotrophic lateral sclerosis in a database population. Validation of a scoring system and a model for survival prediction. Brain 118 (Pt 3): 707–719. 10.1093/brain/118.3.707 7600088

[11] Atsuta N, Watanabe H, Ito M, Tanaka F, Tamakoshi A, et al (2009) Age at onset influences on wide-ranged clinical features of sporadic amyotrophic lateral sclerosis. J Neurol Sci 276: 163–169. 18962725 10.1016/j.jns.2008.09.024

[12] Wilson AC, Dugger BN, Dickson DW, Wang DS (2011) TDP-43 in aging and Alzheimer’s disease - a review. Int J Clin Exp Pathol 4: 147–155. 21326809 PMC3037200

[13] Nakashima-Yasuda H, Uryu K, Robinson J, Xie SX, Hurtig H, et al (2007) Co-morbidity of TDP-43 proteinopathy in Lewy body related diseases. Acta Neuropathol 114: 221–229. 17653732 10.1007/s00401-007-0261-2

[14] Kawahara Y, Kwak S, Sun H, Ito K, Hashida H, et al (2003) Human spinal motoneurons express low relative abundance of GluR2 mRNA: an implication for excitotoxicity in ALS. J Neurochem 85: 680–689. 12694394 10.1046/j.1471-4159.2003.01703.x

[15] Kawahara Y, Ito K, Sun H, Kanazawa I, Kwak S (2003) Low editing efficiency of GluR2 mRNA is associated with a low relative abundance of ADAR2 mRNA in white matter of normal human brain. Eur J Neurosci 18: 23–33. 12859334 10.1046/j.1460-9568.2003.02718.x

[16] Paschen W, Hedreen J, Ross C (1994) RNA editing of the glutamate receptor subunits GluR2 and GluR6 in human brain tissue. J Neurochem 63: 1596–1602. 7523595 10.1046/j.1471-4159.1994.63051596.x

[17] Nishimoto Y, Yamashita T, Hideyama T, Tsuji S, Suzuki N, et al (2008) Determination of editors at the novel A-to-I editing positions. Neurosci Res 61: 201–206. 18407364 10.1016/j.neures.2008.02.009

[18] Kwak S, Nishimoto Y, Yamashita T (2008) Newly identified ADAR-mediated A-to-I editing positions as a tool for ALS research. RNA Biol 5: 193–197. 18971634 10.4161/rna.6925

[19] Lee RH, Heckman CJ (1998) Bistability in spinal motoneurons in vivo: systematic variations in persistent inward currents. J Neurophysiol 80: 583–593. 9705452 10.1152/jn.1998.80.2.583

[20] Lee RH, Heckman CJ (1998) Bistability in spinal motoneurons in vivo: systematic variations in rhythmic firing patterns. J Neurophysiol 80: 572–582. 9705451 10.1152/jn.1998.80.2.572

[21] Cotel F, Antri M, Barthe JY, Orsal D (2009) Identified ankle extensor and flexor motoneurons display different firing profiles in the neonatal rat. J Neurosci 29: 2748–2753. 19261869 10.1523/JNEUROSCI.3462-08.2009 PMC6666223

[22] Riedmann EM, Schopoff S, Hartner JC, Jantsch MF (2008) Specificity of ADAR-mediated RNA editing in newly identified targets. Rna 14: 1110–1118. 18430892 10.1261/rna.923308 PMC2390793

[23] Dengler R, Konstanzer A, Kuther G, Hesse S, Wolf W, et al (1990) Amyotrophic lateral sclerosis: macro-EMG and twitch forces of single motor units. Muscle Nerve 13: 545–550. 2366827 10.1002/mus.880130612

[24] Sturrock RR (1988) Loss of neurons from the motor nucleus of the facial nerve in the ageing mouse brain. J Anat 160: 189–194. 3253254 PMC1262061

[25] Sturrock RR (1991) Stability of motor neuron number in the oculomotor and trochlear nuclei of the ageing mouse brain. J Anat 174: 125–129. 2032929 PMC1256048

[26] Saxena S, Cabuy E, Caroni P (2009) A role for motoneuron subtype-selective ER stress in disease manifestations of FALS mice. Nat Neurosci 12: 627–636. 19330001 10.1038/nn.2297

[27] Hirofuji C, Ishihara A, Roy RR, Itoh K, Itoh M, et al (2000) SDH activity and cell size of tibialis anterior motoneurons and muscle fibers in SAMP6. NeuroReport 11: 823–828. 10757527 10.1097/00001756-200003200-00033

[28] Hashizume K, Kanda K, Burke RE (1988) Medial gastrocnemius motor nucleus in the rat: age-related changes in the number and size of motoneurons. J Comp Neurol 269: 425–430. 3372722 10.1002/cne.902690309

[29] Kanda K, Hashizume K (1989) Changes in properties of the medial gastrocnemius motor units in aging rats. J Neurophysiol 61: 737–746. 2656932 10.1152/jn.1989.61.4.737

[30] Kadhiresan VA, Hassett CA, Faulkner JA (1996) Properties of single motor units in medial gastrocnemius muscles of adult and old rats. J Physiol 493 (Pt 2): 543–552. 10.1113/jphysiol.1996.sp021402 PMC1158936 8782115

[31] Levanon EY, Hallegger M, Kinar Y, Shemesh R, Djinovic-Carugo K, et al (2005) Evolutionarily conserved human targets of adenosine to inosine RNA editing. Nucleic Acids Res 33: 1162–1168. 15731336 10.1093/nar/gki239 PMC549564

[32] Burnashev N, Monyer H, Seeburg P, Sakmann B (1992) Divalent ion permeability of AMPA receptor channels is dominated by the edited form of a single subunit. Neuron 8: 189–198. 1370372 10.1016/0896-6273(92)90120-3

[33] Paschen W, Djuricic B (1995) Regional differences in the extent of RNA editing of the glutamate receptor subunits GluR2 and GluR6 in rat brain. J Neurosci Method 56: 21–29. 10.1016/0165-0270(94)00085-u 7715242

[34] Kawahara Y, Ito K, Sun H, Ito M, Kanazawa I, et al (2004) Regulation of glutamate receptor RNA editing and ADAR mRNA expression in developing human normal and Down’s syndrome brains. Dev Brain Res 148: 151–155. 14757529 10.1016/j.devbrainres.2003.11.008

[35] Nicholas A, de Magalhaes JP, Kraytsberg Y, Richfield EK, Levanon EY, et al (2010) Age-related gene-specific changes of A-to-I mRNA editing in the human brain. Mech Ageing Dev 131: 445–447. 20538013 10.1016/j.mad.2010.06.001 PMC2915444

[36] Van Den Bosch L, Schwaller B, Vleminckx V, Meijers B, Stork S, et al (2002) Protective effect of parvalbumin on excitotoxic motor neuron death. Exp Neurol 174: 150–161. 11922657 10.1006/exnr.2001.7858

[37] Ince P, Stout N, Shaw P, Slade J, Hunziker W, et al (1993) Parvalbumin and calbindin D-28k in the human motor system and in motor neuron disease. Neuropathol Appl Neurobiol 19: 291–299. 8232749 10.1111/j.1365-2990.1993.tb00443.x

