# Supplementary figures and images for: Co-Occurrence of TDP-43 Mislocalization with Reduced Activity of an RNA Editing Enzyme, ADAR2, in Aged Mouse Motor Neurons
Source: PLoS One. 2012 Aug 20;7(8):e43469. doi: 10.1371/journal.pone.0043469 (PMC3423340; doi:10.1371/journal.pone.0043469)

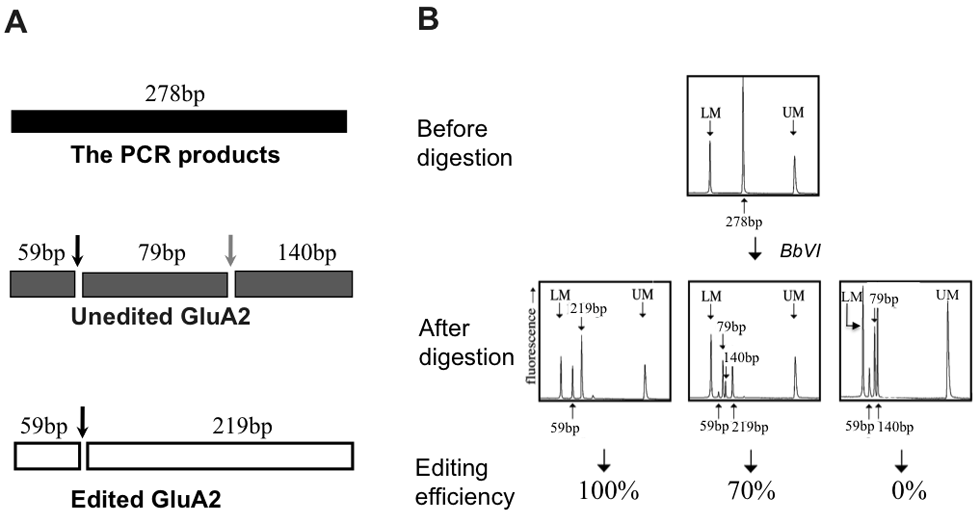

Supplement: Figure S1 — Analysis with a 2100 Bioanalyzer and examples of chromatogram. (A) The PCR products originating from Q/R site-edited GluA2 mRNA had one intrinsic restriction enzyme recognition site (black arrow), whereas those originating from unedited mRNA had an additional recognition site (gray arrow). (B) Restriction digestion of the PCR products originating from edited GluA2 mRNA should produce different numbers of fragments (two bands at 219-bp and 59-bp) from those originating from unedited GluA2 mRNA (three bands at 140-bp, 79-bp and 59-bp). As the 59-bp band would originate from both edited and unedited mRNA, but the 219-bp band would originate from only edited mRNA, we quantified the molarity of the 219- and 59-bp bands using the 2100 Bioanalyzer (Agilent technologies Japan, Tokyo) and calculated the editing efficiency as the ratio of the former to the latter for each sample. (TIF) [file pone.0043469.s001.tif]

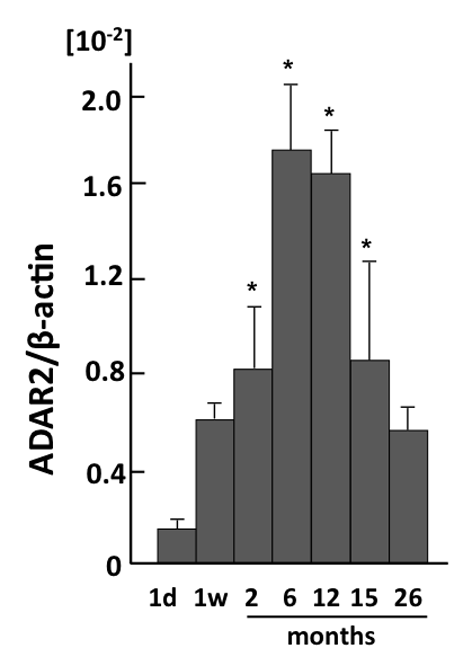

Supplement: Figure S2 — Age-related changes in ADAR2 activity in the AHs. The relative abundance of the ADAR2 mRNA in anterior horns (AHs) is shown at the β-actin mRNA base (n = 3 for each age). The expression level of ADAR2 mRNA developmentally increases and decreases in an age-dependent manner from 26 months of age. The columns and bars represent means ± SEMs. The data were analyzed using repeated ANOVA. Contrast tests were used to compare the means of the ADAR2 activities between the groups after 2 months of age. The values in the four groups (2, 6, 12, and 15 months of age) were significantly higher than the value in the group at 26 months of age (*p<0.01). (TIF) [file pone.0043469.s002.tif]
