# Supplementary material for: Co-Occurrence of TDP-43 Mislocalization with Reduced Activity of an RNA Editing Enzyme, ADAR2, in Aged Mouse Motor Neurons
Source: PLoS One. 2012 Aug 20;7(8):e43469. doi: 10.1371/journal.pone.0043469 (PMC3423340; doi:10.1371/journal.pone.0043469)
Supplement: Table S1 — Primers for PCR, RT-PCR and restriction digestion of PCR products. (DOC) [file pone.0043469.s003.doc]

**SUPPORTINF INFORMATION**

**Table S1**. Primers for PCR, RT-PCR and restriction digestion of PCR products

| **Sequence of primers used for PCR and RT-PCR** | | **Amplified product length (bp)** | **Restriction digestion** |
| --- | --- | --- | --- |
| GluR2 mRNA (Accession no. NM_001039195, NM_001083806) Q/R site | | 278 | Enz: *BbvI*  Ed: 219, 59  Uned: 140, 79, 59  Eff: 219/59 |
| Forward primer | 5’-AGCAGATTTAGCCCCTACGAG-3’ |
| Reverse primer | 5’-CAGCACTTTCGATGGGAGACAC-3’ |
| CYFIP2 mRNA (Accession no. BC056974) K/E site | | 356 | Enz: *MseI*  Ed: 210, 146  Uned: 210, 61, 85  Eff: 146/210 |
| Forward primer | 5’- AAGTGATCCCGGGCTATG -3’ |
| Reverse primer | 5’- GCTCACAGATGTTGTACTGG -3’ |

Enz, restriction enzyme; Ed, length (bp) of restriction digests of PCR products from edited mRNA; Uned, length (bp) of restriction digests of PCR products from unedited mRNA; Eff, bands used for the calculation of the editing efficiency.

|  | |  |
| --- | --- | --- |
|  | |  |
|  |  |
|  |  |
|  | |  |
|  |  |
|  |  |

Table S3. Probes and primers for Real-Time PCR

|  | |  |
| --- | --- | --- |
|  | |  |
|  |  |  |
|  |  |
|  |  |
|  |
|  | |  |
|  |  |  |
|  |  |
|  |  |
|  |
