# Supplementary material for: Co-Occurrence of TDP-43 Mislocalization with Reduced Activity of an RNA Editing Enzyme, ADAR2, in Aged Mouse Motor Neurons
Source: PLoS One. 2012 Aug 20;7(8):e43469. doi: 10.1371/journal.pone.0043469 (PMC3423340; doi:10.1371/journal.pone.0043469)
Supplement: Table S2 — Internal standard for quantitative PCR. (DOC) [file pone.0043469.s004.doc]

**SUPPORTINF INFORMATION**

**Table S2. Internal standard for quantitative PCR**

| **Oligonucleotide sequence** | | **Amplified product length (bp)** |
| --- | --- | --- |
| GluA2 ( Accession no. NM_001039195, NM_001083806) | | 1075 |
| Forward primer | 5’- TCCTTCATCACACCAAGCTTC -3’ |
| Reverse primer | 5’- CCCCAACAATAGTCAGCTTGT -3’ |
| ADAR2 (Accession no. (001024840, AF403109)) | | 1068 |
| Forward primer | 5’- CCACCGCTCTACACTCTCAAC -3’ |
| Reverse primer | 5’- GTGCCAAGGTCACACCGTATC -3’ |

To prepare an internal standard for quantitative PCR, gene-specific PCR products of more than 1 kb in length were amplified from human cerebellar cDNA with the above primers.
