# Supplementary material for: Co-Occurrence of TDP-43 Mislocalization with Reduced Activity of an RNA Editing Enzyme, ADAR2, in Aged Mouse Motor Neurons
Source: PLoS One. 2012 Aug 20;7(8):e43469. doi: 10.1371/journal.pone.0043469 (PMC3423340; doi:10.1371/journal.pone.0043469)
Supplement: Table S3 — Probes and primers for Real-Time PCR. (DOC) [file pone.0043469.s005.doc]

**SUPPORTINF INFORMATION**

Table S3. Probes and primers for Real-Time PCR

| **Oligonucleotide sequence** | | **Amplified product length (bp)** |
| --- | --- | --- |
| GluA2 (Accession no. NM000826, BC028736) | | 146 |
| Forward primer | 5’-GGTCATCAATGCCACAAC -3’ |  |
| Reverse primer | 5’-GGTATGTGGTAGGCTTAGTGA-3’ |  |
| Hybridization probes | 5’-LCRed640-GATGGGCGGCCAAGCTCATCCTT-P-3’ |  |
|  | 5’-AGTACAGCGCGTGCTTACACAGGCG-FITC-3’ |  |
| ADAR2 (Accession no. NM001024840) | | 146 |
| Forward primer | 5’- GGTCATCAATGCCACAAC-3’ |  |
| Reverse primer | 5’- GGTATGTGGTAGGCTTAGTGA -3’ |  |
| Hybridization probes | 5’-LCRed640-GATGGGCGGCCAAGCTCATCCTT-P-3’ |  |
|  | 5’-AGTACAGCGCGTGCTTACACAGGCG-FITC-3’ |  |
